# Effects of Tai-Chi Chuan Practice on Patterns and Stability of Lower Limb Inter-Joint Coordination During Obstructed Gait in the Elderly

**DOI:** 10.3389/fbioe.2021.739722

**Published:** 2021-12-21

**Authors:** Chien-Chung Kuo, Sheng-Chang Chen, Jr-Yi Wang, Tsung-Jung Ho, Jaung-Geng Lin, Tung-Wu Lu

**Affiliations:** ^1^ Department of Orthopedics, School of Medicine, China Medical University, Taichung, Taiwan; ^2^ Department of Orthopedics, China Medical University Hospital, Taichung, Taiwan; ^3^ Department of Biomedical Engineering, National Taiwan University, Taipei, Taiwan; ^4^ Department of Orthopedics, Shuang Ho Hospital, Taipei Medical University, Taipei, Taiwan; ^5^ Integration Center of Traditional Chinese and Modern Medicine, Buddhist Tzu Chi General Hospital, Hualien, Taiwan; ^6^ Department of Chinese Medicine, Buddhist Tzu Chi General Hospital, Taipei, Taiwan; ^7^ School of Post-Baccalaureate Chinese Medicine, Tzu Chi University, Hualien, Taiwan; ^8^ Institute of Chinese Medical Science, China Medical University, Taichung, Taiwan

**Keywords:** gait analysis, obstacle crossing, phase plot, dynamic systems, coordination

## Abstract

Losing balance or tripping during obstacle-crossing is one of the most frequent causes of falls in the elderly. As a low speed, low impact exercise, Tai Chi Chuan (TCC) can be promising in helping the elderly develop strategies for improved balance, inter-joint coordination, and end-point control during obstacle-crossing. This study investigates the effects of TCC training on the patterns and variability of the lower-limb inter-joint coordination during obstacle-crossing in the elderly. Fifteen older TCC practitioners and 15 healthy controls crossed obstacles of three different heights, while sagittal angles (x) and angular velocities $\left(x^{\prime }\right)$ of the hips, knees and ankles were measured and their phase angles obtained. The continuous relative phases (CRP) of the hip-knee and knee-ankle coordination were also calculated. The standard deviations of the CRP curve points were averaged to obtain deviation phase (DP) values for the stance and swing phases. The TCC group was found to cross obstacles with increased leading and trailing toe-clearances with unaltered CRP values when the swing toe was above the obstacle. Long-term TCC training altered the patterns and magnitudes of the CRPs primarily over double-limb support and significantly reduced the variabilities of leading knee-ankle and trailing hip-knee and knee-ankle CRP curves over the crossing cycle, regardless of obstacle height. The current results suggest that long-term TCC practice was helpful for a crossing strategy with significantly increased foot-obstacle clearances and reduced variability of the way the motions of the lower limb joints are coordinated during obstacle-crossing. These benefits may be explained by the long-lasting effects of continuous practice of the slow movement patterns emphasizing between-limb transfer of body weight in TCC.

## Introduction

More than 30% of people aged 65 and over fall at least once during a year, with a higher incidence of falls in frail individuals or those with disabilities (Blake et al., 1988; Tinetti et al., 1988; Campbell et al., 1990; Graafmans et al., 1996). Many older people, even those who are not injured by the fall, develop a fear of falling (Bhala et al., 1982; Tinetti et al., 1994). This fear may cause them to limit their activities, leading to reduced mobility and physical fitness, thus increasing their actual risk of falling (Vellas et al., 1997). Among the causes of falls in the elderly, losing balance or tripping during obstacle-crossing is one of the most frequent (Overstall et al., 1977; Blake et al., 1988; Tinetti and Speechley, 1989; Campbell et al., 1990; Winter, 1992; McFadyen and Prince, 2002; Di Fabio et al., 2004). Obstacle-crossing during walking is a complex motor task, requiring precise end-point (swing foot) control while maintaining body balance via highly coordinated joint movements (Chen et al., 2004; Chen and Lu, 2006; Lu et al., 2006). Maintenance of the body’s balance, together with well-coordinated joint movement and sufficient foot-obstacle clearance of the swing limb, are essential for successful obstacle-crossing (Chen et al., 2004; Chen et al., 2008; Lu et al., 2010). On the other hand, inappropriate control of the joints of the locomotor system may contribute to body imbalance which may further lead to tripping over obstacles (Chen et al., 1994; Chou and Draganich, 1998; Draganich and Kuo, 2004; McKenzie and Brown, 2004; Lu et al., 2006; Liu et al., 2010; Hsu et al., 2016). There is a need for interventions to help the elderly develop strategies for improved balance, inter-joint coordination, and end-point control during obstacle-crossing. A promising candidate is Tai-Chi Chuan (TCC). As a low speed, low impact exercise, TCC has been shown to be beneficial for retaining or regaining proper balance and coordination for older people (Lai et al., 1995; Wong et al., 2001), including improved balance control during obstacle-crossing (Kim, 2009). However, few studies have explored the benefits of TCC practice on lower limb inter-joint coordination and end-point control during obstacle-crossing in the elderly.

TCC is an ancient Chinese martial art that consists of a series of slow, continuous and gentle motions transitioning from double-limb to single-limb support, thus focusing on dynamic weight-shifting to a narrower base of support. TCC is also a coordination exercise that is helpful for maintaining the ability of posture control to reduce the risk of falling (Wong et al., 2001; Wong et al., 2009; Burschka et al., 2014; Kong et al., 2019). Long-term TCC practice appeared to be helpful for attenuating the age-related decline in general physical function, and thus a suitable exercise for the elderly to modify their gait and movement patterns (Wolf et al., 1997; Lin et al., 2006; Mak et al., 2017). Older people with long-term TCC experience have been shown to have increased muscle strength (Jacobson et al., 1997; Lan et al., 2000; Wu et al., 2002b), balance (Wu, 2002; Wu et al., 2002b; Mak and Ng, 2003; Tsang and Hui-Chan, 2004), inter-joint coordination (Wang et al., 2010) and sensory organization in postural control (Wong et al., 2001; Tsang et al., 2004). These changes are all important components for preventing falls in the elderly (Tse and Bailey, 1992; Wolfson et al., 1996; Wolf et al., 1997; Hass et al., 2004), providing some explanation for the observed reduction in falls in older people with TCC training (Hass et al., 2004; Choi et al., 2005; Mak et al., 2017). Apart from the benefits on general physical function (Birimoglu Okuyan and Bilgili, 2017; Liu et al., 2019; Birimoglu Okuyan and Deveci, 2021), previous studies have also shown the effects of TCC training on posture, gait and movement performance, such as standing balance (Ho et al., 2012), walking performance (Hackney and Earhart, 2008; Li et al., 2012; Li et al., 2014). However, previous studies on the effects of TCC training on obstacle-crossing performance in the elderly are limited. Only a limited number of studies investigated the effects of TCC on temporospatial parameters and balance control during obstacle-crossing (Ramachandran et al., 2007; Kim, 2009; Kim et al., 2013; Chang et al., 2015). It remains unclear whether TCC training would improve the lower limb inter-joint coordination and end-point control critical for a safe and successful obstacle-crossing in the elderly.

Computerized gait analysis has been used to identify the effects of ageing on the end-point and joint kinematics during obstacle-crossing in terms of variables such as foot-obstacle clearances, foot-obstacle distances, and joint angles and moments (Lu et al., 2006; Lu et al., 2007; Yen et al., 2009). Although considering the human pelvis-leg apparatus as a multi-link system enabled the synthesis of the kinematic changes of individual joints and endpoints to identify the kinematic strategies of obstacle-crossing in various populations (Hsu et al., 2016; Chien and Lu, 2017; Wu et al., 2019), such approach did not reveal multi-joint coordination performance (Lu et al., 2006). Inter-joint coordination is the relationship between the motions of two joints, including angular positions and velocities associated with the efferent motor control and information from afferent joint receptors (Burgess-Limerick et al., 1993). To gain insight into the control strategies of obstacle-crossing, relative phase analysis has been used to study the patterns and variability of multi-joint coordination during the motor task in various subject groups (Lu et al., 2008; Wang et al., 2009; Yen et al., 2009). Among the methods previously proposed to quantify inter-joint coordination, only the technique of relative phase plots combines information on joint angular positions and velocities (Burgess-Limerick et al., 1993; Cirstea et al., 2003). While relative phases between two joints describe the patterns of the coupling of the joints (Burgess-Limerick et al., 1993), the variability of the relative phase plots of repeated trials quantifies the stability of the inter-joint coordination during multi-joint movements (Burgess-Limerick et al., 1993; Hamill et al., 1999; van Uden et al., 2003). Although with altered kinematics and kinetics in individual joints compared to young adults (Chen et al., 1991; Lu et al., 2006; Weerdesteyn et al., 2006), older adults did not change the way the lower limb joints were coordinated (i.e., inter-joint coordination patterns) except with a larger variability during obstacle-crossing (Yen et al., 2009), suggesting that age-related changes contributed mainly to reduced stability of the lower limb inter-joint coordination during obstacle-crossing (Burgess-Limerick et al., 1993; Hamill et al., 1999; van Uden et al., 2003). Therefore, quantifications of the patterns and variabilities are equally important in the studies of the effects of TCC training on lower limb inter-joint coordination during obstacle-crossing in the elderly, which has not been reported in the literature.

The purpose of this study was to determine the effects of long-term TCC practice on the patterns and variability of the inter-joint coordination of the lower extremities in the elderly during obstacle-crossing. It was hypothesized that older people who had practised TCC over a long period of time and those without TCC experience would show similar lower limb inter-joint coordination patterns but with different magnitudes and variabilities during obstacle-crossing, regardless of obstacle height.

## Materials and Methods

### Ethics Statement

All experiments of the current study were conducted under the approval of China Medical University Hospital Institutional Review Board (IRB No. DMR98-IRB-072). All the experiments and procedures conformed to the Ethical Principles for Medical Research Involving Human Subjects (World Medical Association Declaration of Helsinki). All participants were informed of the procedure, and they provided written informed consent prior to the study.

### Subjects

Fifteen older adults who had practised TCC continuously for at least an hour a day and for 5 days a week for more than 13 years (TCC group, gender: 4 females/11 males, age: 72 ± 5.5 years, height: 164 ± 6.1 cm, mass: 57.7 ± 7.5 kg, TCC experience: 22 ± 10.1 years), and 15 healthy controls who had done leisure exercises (walking or jogging) for the same time period, and matched with the TCC group for sex, age and BMI (Control group, gender: 4 females/11 males, age: 71 ± 6.1 years, height: 161 ± 5.7 cm, mass: 62 ± 10.9 kg) participated in the current study. They were free of any neuromusculoskeletal dysfunction and with normal corrected vision.

### Experimental Protocol

**FIGURE 1:**
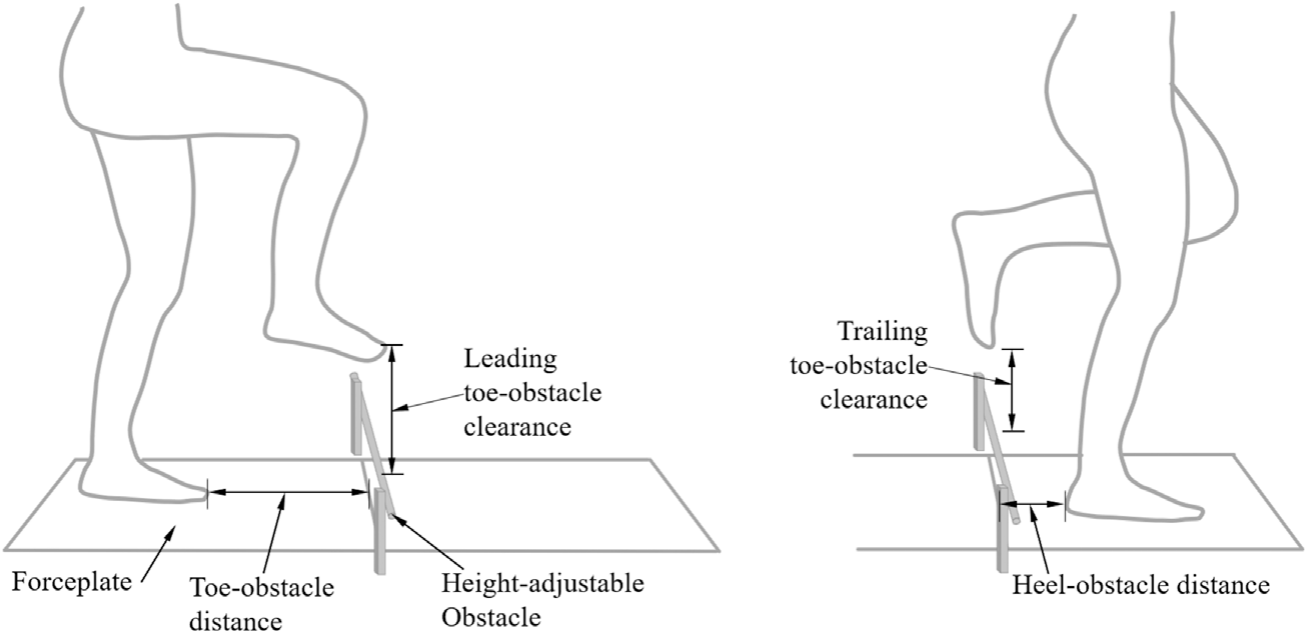
Schematic descriptions of the experimental setup. Two forceplates are placed on either side of a height-adjustable obstacle. The leading and trailing toe-obstacle clearances, toe-obstacle and heel-obstacle distances are also indicated.

In a university hospital gait laboratory, each of the subjects walked at their preferred walking speed along a 10-m walkway and crossed a tube-like obstacle placed horizontally across a height-adjustable frame for three different obstacle heights, i.e., 10, 20 and 30% of the subject’s leg length (Chen and Lu, 2006). The position of the obstacle was defined by two infrared retro-reflective markers placed on either end of the tube (Figure 1), while the motions of the body segments were tracked by another 39 infrared-retroreflective markers placed on the ASISs, PSISs, greater trochanters, mid-thighs, medial and lateral epicondyles, heads of fibulae, tibial tuberosities, medial and lateral malleoli, navicular tuberosities, fifth metatarsal bases, big toes and heels, and mandibular condylar processes, acromion processes, C7, medial and lateral humeral epicondyles, and ulnar styloid (Hong et al., 2015). Three-dimensional trajectories of the markers were measured using a seven-camera motion capture system (Vicon 512, Oxford Metrics Group, United Kingdom) at 120 Hz, while two forceplates (AMTI, United States) placed on either side of the obstacle were used to measure the ground reaction forces at 1,080 Hz. Marker and forceplate data of three complete crossing trials for each lower limb leading were obtained for each obstacle height. The sequence of the obstacle-height conditions for each subject was decided by a random number table based on a counterbalanced measures design. The desired lead limb was elicited by changing the starting position of the subject.

### Data Analysis

For the quantification of the patterns and variability of the inter-joint coordination of the lower extremities, the pelvis-leg apparatus was modeled as a system of seven rigid segments, namely the pelvis, thighs, shanks and feet (Lu et al., 2007; Liu et al., 2010; Chen et al., 2015). Each body segment was embedded with an orthogonal coordinate system with the positive x-axis directed anteriorly, positive y-axis superiorly and positive z-axis to the right following the ISB recommendations (Wu et al., 2002a). Soft tissue artifacts of the markers were reduced using a global optimization method that minimized the weighted sum of squared distances between measured and calculated marker positions (Lu and O’connor, 1999). The angles of each joint were calculated from the rotation matrix of the associated distal segment relative to the proximal using a Cardanic rotation sequence (z–x–y) (Cole et al., 1993). The time-history of each lower-limb joint angle in the sagittal plane over the crossing stride (CS) was fitted by a quintic spline using the GCVSPL method (Woltring, 1986) to calculate the corresponding angular velocities (Lu et al., 2008). The CS of the leading limb began at toe-off before the obstacle and ended at toe-off after clearing the obstacle, while that for the trailing limb was defined as heel-strike before the obstacle to heel-strike after clearing the obstacle. The events of heel-strike and toe-off were determined according to a 10N-threshold for the GRF or by a foot marker-based algorithm (Ghoussayni et al., 2004). Crossing speed was also calculated as the average speed of the mid-point of the inter-ASIS line in the direction of progression over the crossing stride (Lu et al., 2007). Toe-obstacle and heel-obstacle clearances were calculated as the vertical distances between the obstacle and toe and heel markers, respectively, when the corresponding marker was above the obstacle (Figure 1). Trailing toe-obstacle distances and leading heel-obstacle distances were also calculated as the horizontal distances between the obstacle and toe and heel markers, respectively, when the corresponding foot was on the floor (Figure 1; Liu et al., 2010). Joint angular displacements were normalized such that the ranges of the joint angles during a CS lay between −1 and 1, with the midpoint located at zero. Joint angular velocities were normalized by the maximum absolute velocity over the CS, maintaining zero velocity as zero (Li et al., 1999; Stergiou et al., 2001a). These normalization procedures helped minimize the influence of different movement amplitudes and frequencies (Peters et al., 2003). Phase plots of normalized angular velocities $\left(x^{\prime }\right)$ against normalized angular displacements (x) for each joint were generated, and the phase angles (φ) were calculated in the Euclidean plane as the angle between the positive x-axis and the ray to the point (x, x’) using the 2-argument arctangent function. Any discontinuities between consecutive angles greater than or equal to π were corrected by adding multiples of ±2π until the jump was less than π. Continuous relative phases (CRP), representing the coordination between two adjacent joints, were then calculated by subtracting the phase angle of the distal joint from that of the proximal at the same time instant (Burgess-Limerick et al., 1993). In the current study, the CRP curves for the inter-joint coordination between hip and knee $\left(\varphi_{hip-knee}\right)$ and between knee and ankle $\left(\varphi_{knee-ankle}\right)$ were obtained. A positive CRP indicates that the proximal joint leads the distal, while a negative one means the reverse. The two adjacent joints are moving in a similar fashion or in-phase if the CRP is close to 0° or 360°, and are moving in an opposite fashion or out-of-phase if the CRP is close to 180°. For each inter-joint relationship, the CRP curves from the six trials of all subjects were ensemble-averaged to reveal the general patterns. Coefficients of multiple correlation (CMC) (Kadaba et al., 1989; Silfies et al., 2009), were also calculated to quantify the similarity of the CRP patterns between the TCC and healthy control groups. If the patterns are similar, the CMC values are close to unity. The between-group differences of the ensemble-averaged CRP curves were quantified by the root-mean-squared differences (RMSD). The CMC gave temporal differences in the phase shift while the RMSD reported differences in the magnitude and changes in the patterns of relative phases (Chiu et al., 2010). A high cross-correlation coefficient with a low RMS difference would indicate that the two curves are similar, both in pattern and magnitude (Haddad et al., 2006). To indicate the variability of each inter-joint coordination relationship, a parameter called deviation phase (DP) was calculated by first calculating the standard deviation of each point on the ensemble curve, and then averaging all the standard deviations over the profile for the whole crossing cycle, and single-limb support (SLS), swing, and double-limb support (DLS) phases (Stergiou et al., 2001b). A low DP value indicates a less variable relationship between the two joints (Stergiou et al., 2001b).

### Statistical Analysis

The preferred crossing speed, leading and trailing toe-obstacle and heel-obstacle clearances, and the CRP values at the instance when the swing toe was above the obstacle, as well as DP variables were analyzed using a two-way mixed-design analysis of variance (ANOVA) with one between-subject factor (group) and one within-subject factor (obstacle height). If there were no interactions between the factors, the main effects would be reported. A *post hoc* trend analysis was performed to determine the linear trend if a height effect was found. If a significant group effect on the crossing speed was found, a 2 × 1 one-way analysis of covariance (ANCOVA) with crossing speed as a covariate would be performed to remove the confounding effect of crossing speed. If there were significant interactions between the main factors, pair-wise between-group comparisons were performed using an independent t-test for each obstacle height, and a *post hoc* trend analysis was performed to determine the linear trend for each group. The significance level was set at *α* = 0.05 for all tests. All statistical analyses were conducted using SPSS (Version 13.0, Chicago, IL).

An *a priori* power analysis based on pilot results of the DP values of the hip-knee and knee-ankle CRP curves from four subjects for each group using G*POWER (Erdfelder et al., 1996) for a two-way mixed-design ANOVA with one between-subject factor (group) and one within-subject factor (obstacle height) determined that a projected sample size of 10 subjects for each group would be needed with a power of 0.8 and large effect size (Cohen’s d = 0.78) at a significance level of 0.05. A sample size of 15 subjects for each group was selected in the current study (Wang et al., 2009; Chiu et al., 2010).

## Results

The TCC group showed significantly increased leading toe-obstacle and heel-obstacle clearances and trailing toe-obstacle clearance compared to the Control group (*p* < 0.05, partial h^2^ > 0.2; Table 1). No significant between-group differences were found in crossing speed, trailing heel-obstacle clearance, and toe-obstacle and heel-obstacle distances (Table 1). With increasing obstacle height, the leading toe-obstacle and heel-obstacle clearances and trailing heel-obstacle clearance were increased linearly with linearly decreased heel-obstacle distances and crossing speed (*p* < 0.05, partial h^2^ > 0.12; Table 1).

**TABLE 1:** Means (standard deviations, SD) of the crossing speed, toe-obstacle clearance and horizontal distances in the TCC and Control groups when crossing obstacles of heights of 10, 20 and 30% LL difference.

	Obstacle height (%LL)			Main effects	Partial Eta Squared
	10	20	30		
Crossing speed (m/s)					
TCC	0.70 (0.05)	0.66 (0.08)	0.60 (0.08)	*p*_g_ = 0.12	0.12
Control	0.80 (0.14)	0.71 (0.12)	0.64 (0.11)	*p*_h_ = 0.01↓	0.73
Leading toe-obstacle clearance (mm)					
TCC	183.3 (23.4)	192.5 (24.5)	200.9 (19.1)	*p*_g_ = 0.03^+^	0.23
Control	147.8 (47.8)	169.0 (32.9)	183.8 (42.8)	*p*_h_ = 0.01↑	0.23
Trailing toe-obstacle clearance (mm)					
TCC	173.2 (31.1)	181.1 (41.3)	182.9 (45.7)	*p*_g_ = 0.03^+^	0.22
Control	129.1 (45.6)	133.9 (51.9)	149.4 (55.3)	*p*_h_ = 0.48	0.03
Leading heel-obstacle clearance (mm)					
TCC	147.0 (39.2)	153.2 (35.1)	172.2 (31.5)	*p*_g_ = 0.04^+^	0.20
Control	120.4 (24.5)	133.7 (24.3)	141.4 (31.8)	*p*_h_ = 0.01↑	0.21
Trailing heel-obstacle clearance (mm)					
TCC	340.8 (50.0)	361.2 (45.1)	361.7 (44.6)	*p*_g_ = 0.72	0.01
Control	335.1 (48.5)	344.9 (49.3)	365.8 (73.0)	*p*_h_ = 0.04↑	0.12
Toe-obstacle horizontal distance (% LL)					
TCC	24.9 (4.8)	25.4 (4.8)	24.4 (5.1)	*p*_g_ = 0.86	0.01
Control	24.9 (4.4)	24.6 (4.2)	24.2 (4.7)	*p*_h_ = 0.28	0.05
Heel-obstacle horizontal distance (% LL)					
TCC	17.3 (2.8)	16.4 (3.3)	15.6 (3.5)	*p*_g_ = 0.89	0.01
Control	18.4 (5.1)	16.4 (3.7)	15.1 (4.5)	*p*_h_ = 0.01↓	0.18

No interaction was found between group and obstacle height factors. LL, leg length; p_h_, p-value for obstacle height effect; p_g_, p-value for group effect; ^+^, significant difference between groups; ↑, linearly increasing trend; ↓, linearly decreasing trend.

**FIGURE 2:**
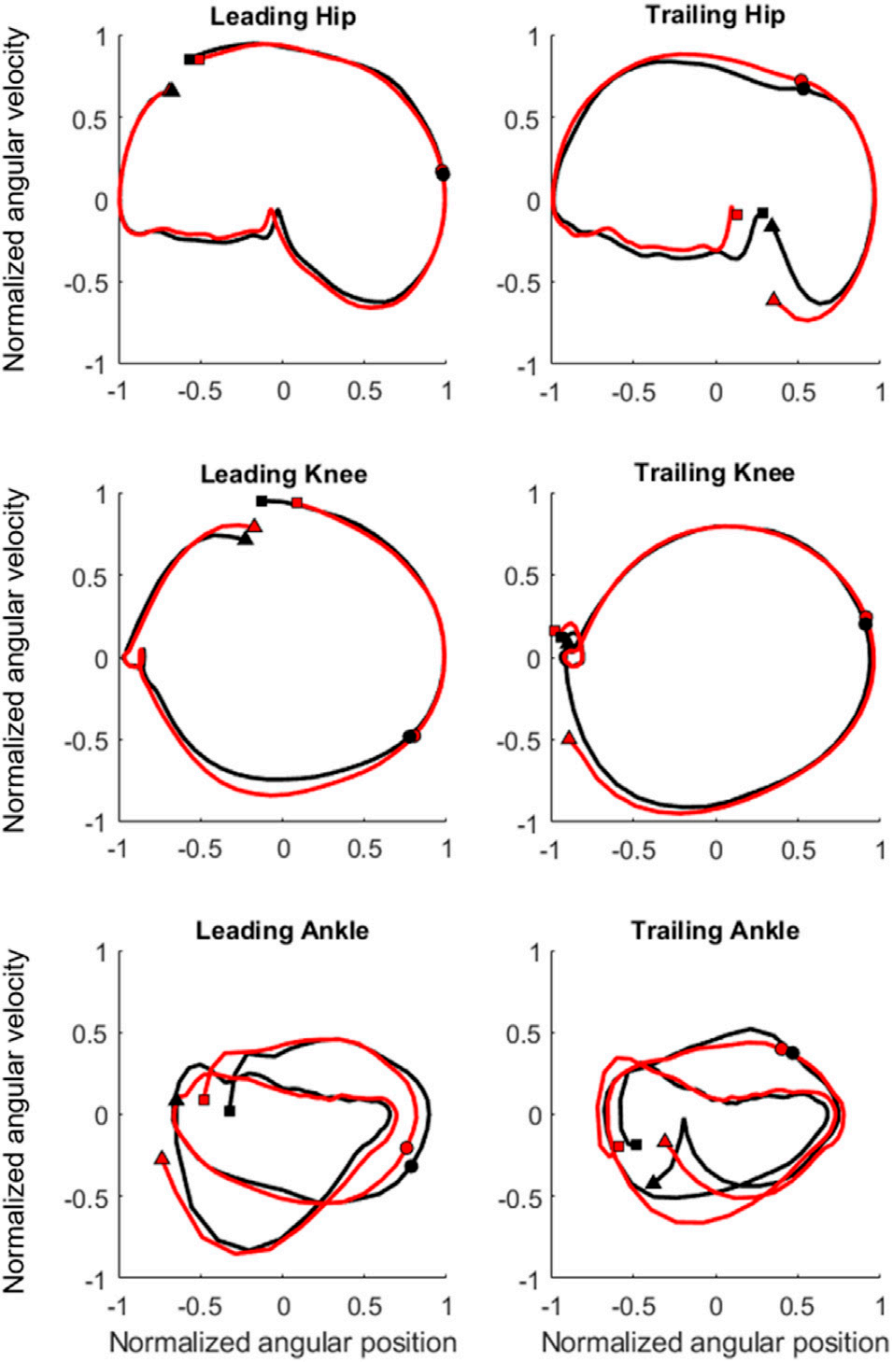
Ensemble-averaged phase plots of the hip, knee and ankle of the leading and trailing limbs for the TCC (red lines) and Control (black lines) groups when crossing obstacles of 20% of leg length. Square markers indicate the beginning of the crossing cycle, circular markers the instance when the toe was above the obstacle, and triangular markers the end of the crossing cycle.

From the ensemble-averaged phase plots of the leading and trailing joints for both groups, the phase trajectories of the hip and knee were found to be in forms of nearly-closed periodical circles, but those for the ankle had a different form of trajectories with varying amplitudes (Figure 2). These trajectories were similar for the hip and knee for all obstacle heights between groups, but between-group differences existed for the ankle joints of both the leading and trailing limbs (Figure 2).

**FIGURE 3:**
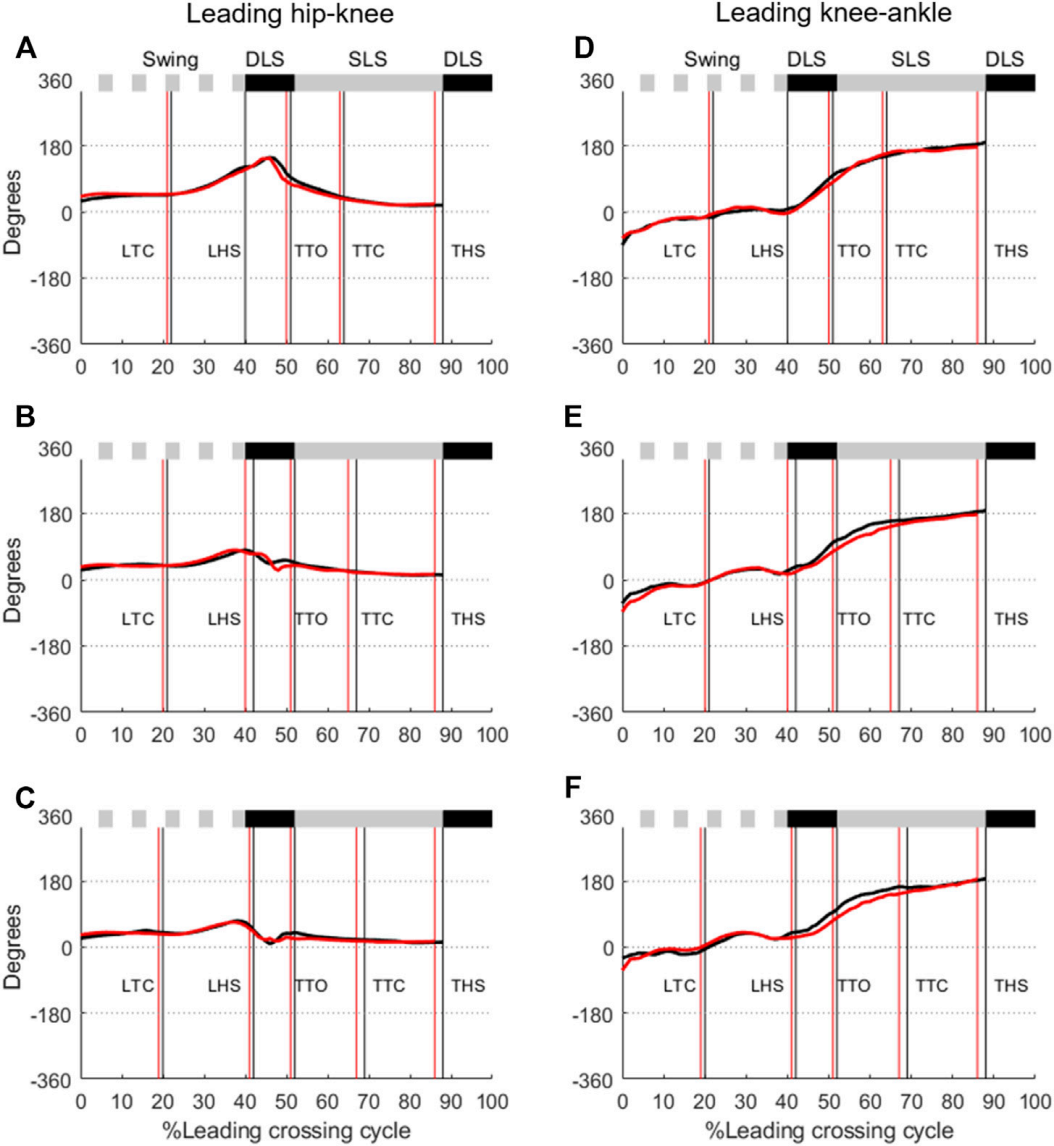
Ensemble-averaged relative phase angles of the leading hip-knee coordination when crossing obstacles of **(A)** 10%, **(B)** 20%, and **(C)** 30% of leg length for the TCC (red lines) and Control (black lines) groups. Corresponding plots for the leading knee-ankle coordination are given in **(D–F)**. (Solid black horizontal bar: double-limb support; Solid gray horizontal bar: single-limb support; Dotted gray horizontal bar: swing phase; LTC, leading toe-crossing; LHS, leading heel-strike; TTO, trailing toe-off; TTC, trailing toe-crossing; THS, trailing heel-strike).

**FIGURE 4:**
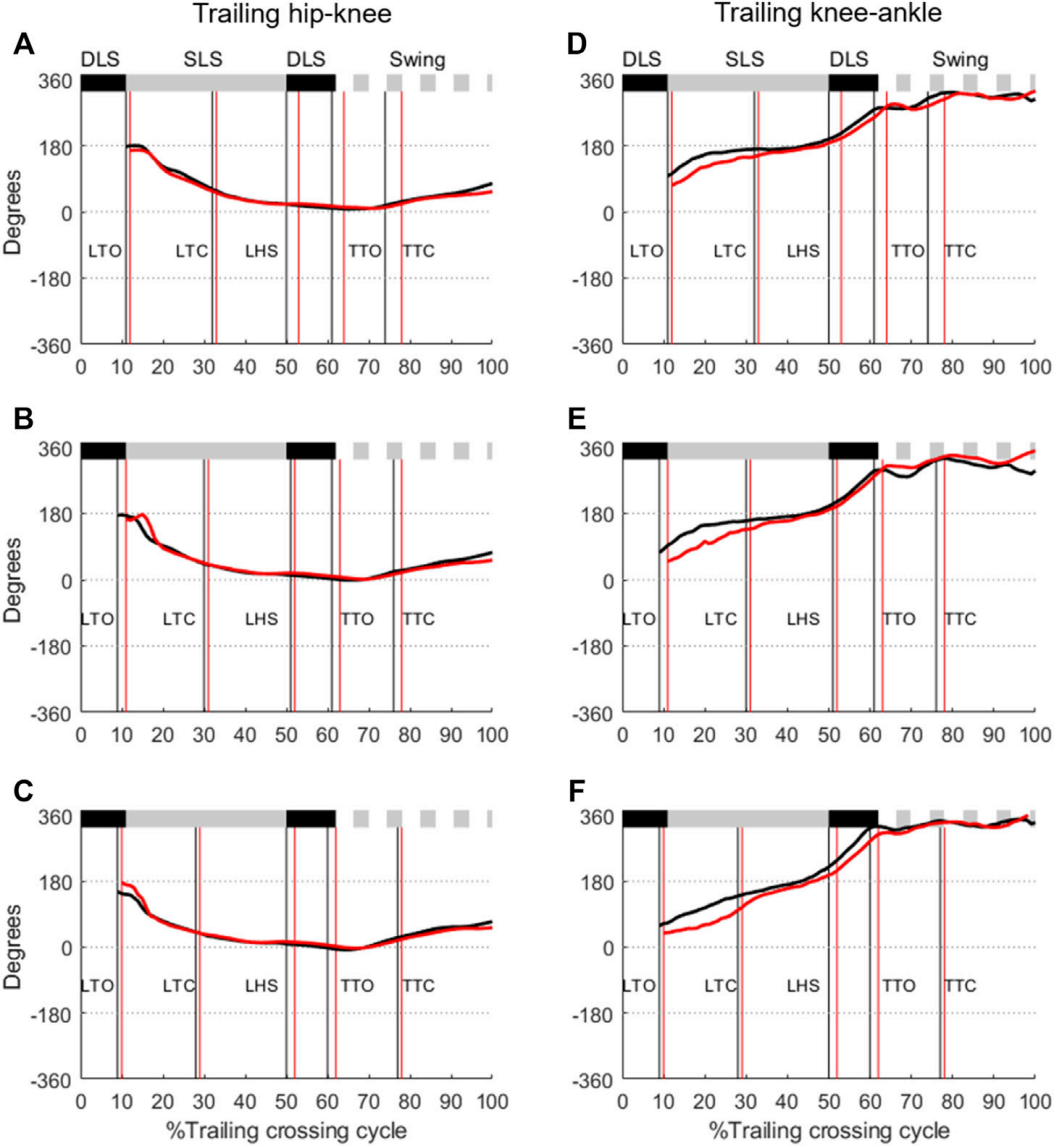
Ensemble-averaged relative phase angles of the trailing hip-knee coordination when crossing obstacles of **(A)** 10%, **(B)** 20%, and **(C)** 30% of leg length for the TCC (red lines) and Control (black lines) groups. Corresponding plots for the trailing knee-ankle coordination are given in **(D–F)**. (Solid black horizontal bar: double-limb support; Solid gray horizontal bar: single-limb support; Dotted gray horizontal bar: swing phase; LTO, leading toe-off; LTC, leading toe-crossing; LHS, leading heel-strike; TTO, trailing toe-off; TTC, trailing toe-crossing).

The ensemble-averaged hip-knee CRP curves of the leading and trailing limbs were retained within the range of 0° and 180° for all obstacle heights and for both groups, while those for knee-ankle coordination were within around 0° and 360° (Figures 3, 4). For the leading-limb crossing stride, the hip-knee CRP curves started from 0° at the beginning of the swing phase, increased smoothly to about 180° at the end of the swing phase, and then returned to 0° at the beginning of the stance phase (Figure 3). The CRP curves for knee-ankle coordination in the leading and trailing limbs remained around 0° (or 360°) during swing phase, and increased from 0° at the beginning of the stance phase to 360° at the end of the stance phase (Figure 3). For the trailing-limb crossing stride, the hip-knee CRP curves started from 0° at the beginning of the swing phase and increased smoothly to about 180° at the end of the swing phase, then returned to 0° at the beginning of the stance phase (Figure 4). The knee-ankle CRP curves of the leading and trailing limbs remained around 0° (or 360°) during swing phase, and increased from 0° at the start of the stance phase to 360° at the end of the stance phase (Figure 4).

No group effect was found for the CRP values of the leading and trailing limbs when the swing toe was above the obstacle (Figures 3, 4; Table 2). For the leading limb, the CMC values of the hip-knee and knee-ankle CRP curves between the TCC and elderly control groups over Swing and SLS phases were all greater than 0.81 (0.81–0.93) for all obstacle-height conditions, but those over the DLS were less than 0.78 (0.39–0.78, Table 3). The between-group RMSD values of the hip-knee CRP curves over DLS ranged from 8.6 to 14.6 in comparison with 3.7–6.5 over Swing and SLS, while the knee-ankle RMSD values greater than 10.0 were found during DLS and SLS. For the trailing limb, the hip-knee CMC values for Swing and SLS phases were greater than 0.93 for all obstacle-height conditions, while those for DLS were less than 0.17 (Table 3). The knee-ankle CMC values over Swing, DLS and SLS phases ranged from 0.10 to 0.75 (Table 3). The RMSD values greater than 10.0 were found for knee-ankle for all obstacle conditions during all phases (Table 3).

**TABLE 2:** Means (standard deviations, SD) of ensemble-averaged CRP of the hip-knee and knee-ankle coordination at instants when the leading toe and the trailing toe were above the obstacle for the TCC and Control groups.

		Obstacle height (%LL)			Main effects	Partial Eta squared
		10	20	30		
Leading toe above the obstacle						
Leading hip-knee	TCC	63.5 (43.2)	76.1 (72.2)	92.3 (111.8)	*p*_g_ = 0.66	0.01
	Control	48.6 (17.7)	53.2 (32.5)	106.7 (89.4)	*p*_h_ = 0.03↑	0.13
Leading knee-ankle	TCC	−36.0 (38.2)	−12.4 (39.5)	0.5 (25.3)	*p*_g_ = 0.53	0.02
	Control	−31.2 (50.8)	−0.8 (49.6)	3.2 (28.7)	*p*_h_ = 0.01↓	0.21
Trailing hip-knee	TCC	24.2 (6.9)	20.7 (6.7)	12.7 (4.3)	*p*_g_ = 0.23	0.06
	Control	28.3 (7.9)	23.0 (7.9)	14.5 (4.3)	*p*_h_ = 0.01↓	0.76
Trailing knee-ankle	TCC	161.8 (29.6)	141.9 (23.5)	133.8 (26.3)	*p*_g_ = 0.87	0.01
	Control	157.6 (30.4)	137.5 (46.2)	146.9 (38.6)	*p*_h_ = 0.01↓	0.15
Trailing toe above the obstacle						
Leading hip-knee	TCC	38.5 (8.9)	27.7 (12.9)	42.5 (84.5)	*p*_g_ = 0.66	0.01
	Control	44.4 (14.5)	27.8 (19.5)	23.6 (5.2)	*p*_h_ = 0.25	0.05
Leading knee-ankle	TCC	144.5 (33.8)	139.4 (42.1)	140.9 (33.6)	*p*_g_ = 0.22	0.06
	Control	149.3 (35.5)	156.6 (39.6)	169.7 (63.4)	*p*_h_ = 0.51	0.03
Trailing hip-knee	TCC	42.7 (5.7)	38.5 (9.4)	42.1 (7.7)	*p*_g_ = 0.26	0.05
	Control	42.9 (11.1)	42.5 (12.3)	49.1 (9.2)	*p*_h_ = 0.11	0.09
Trailing knee-ankle	TCC	307.5 (39.4)	327.2 (54.8)	340.2 (41.1)	*p*_g_ = 0.49	0.02
	Control	325.5 (26.6)	329.5 (37.3)	344.1 (24.7)	*p*_h_ = 0.01↑	0.19

LL, leg length; *p_h_*, *p*-value for obstacle height effect; *p_g_*, *p*-value for group effect; +, significant difference between groups; ↑, linearly increasing trend; ↓, linearly decreasing trend.

**TABLE 3:** Coefficient of multiple correlation (CMC) values and the RMS differences of the CRP curves between TCC and Control groups during obstacle-crossing.

Obstacle height (%LL)	Leading limb			Trailing limb		
	10	20	30	10	20	30
Hip-knee CMC						
Swing	0.92	0.81	0.83	0.93	0.95	0.95
DLS	0.54	0.47	0.78	0.16	0.17	0.10
SLS	0.90	0.85	0.82	0.96	0.97	0.97
Knee-ankle CMC						
Swing	0.93	0.90	0.87	0.6	0.10	0.42
DLS	0.78	0.68	0.39	0.67	0.75	0.51
SLS	0.92	0.81	0.86	0.45	0.34	0.59
Hip-knee RMSD						
Swing	6.0	6.1	6.1	7.9	5.1	5.1
DLS	14.6	13.1	10.4	4.1	4.6	5.6
SLS	6.5	3.7	6.9	7.1	8.3	7.7
Knee-ankle RMSD						
Swing	7.6	8.2	8.8	11.2	18.9	11.5
DLS	13.5	13.9	17.7	16.5	13.2	31.0
SLS	10.4	14.3	15.8	24.0	32.2	32.2

LL, leg length.

For the variability of the inter-joint coordination, the TCC group showed significantly smaller DP values of the hip-knee and knee-ankle CRP curves over all the phases of the crossing stride for both limbs when compared to the Control group (*p* < 0.04, partial h^2^ > 0.21; Table 4), except for the leading hip-knee CRP curves showing no significant between-group differences (*p* > 0.61, partial h^2^ < 0.03; Table 4). During SLS of the trailing limb, the DP values decreased linearly with increasing obstacle height for most CRP curves (*p* < 0.05, partial h^2^ > 0.18; Table 4), except for the knee-ankle of the trailing limb showing an increasing trend (*p* = 0.03, partial h^2^ = 0.20; Table 4).

**TABLE 4:** Means (standard deviations, SD) of the DP values of the CRP curves for the TCC and Control groups.

		Obstacle height (%LL)			Main effects	Partial Eta squared
		10	20	30		
Trailing limb SLS					
Leading hip-knee	TCC	8.4 (3.6)	5.7 (1.9)	5.8 (2.0)	*p*_g_ = 0.79	0.01
	Control	8.3 (6.4)	5.2 (1.8)	5.5 (1.9)	*p*_h_ = 0.01↓	0.29
Leading knee-ankle	TCC	17.2 (6.3)	16.8 (4.8)	11.5 (2.1)	*p*_g_ = 0.01+	0.46
	Control	24.2 (7.7)	18.3 (8.6)	16.0 (6.7)	*p*_h_ = 0.05↓	0.19
Trailing hip-knee	TCC	8.4 (2.9)	6.9 (2.2)	5.8 (2.9)	*p*_g_ = 0.33	0.06
	Control	8.7 (2.5)	7.8 (3.0)	6.5 (1.8)	*p*_h_ = 0.03↓	0.18
Trailing knee-ankle	TCC	12.8 (3.9)	13.5 (5.4)	17.4 (5.8)	*p*_g_ = 0.01+	0.64
	Control	18.6 (5.4)	21.5 (5.2)	23.8 (4.2)	*p*_h_ = 0.03↑	0.20
DLS					
Leading hip-knee	TCC	27.6 (11.2)	20.5 (9.2)	20.7 (7.9)	*p*_g_ = 0.61	0.03
	Control	24.8 (8.5)	23.6 (8.1)	28.2 (24.0)	*p*_h_ = 0.74	0.03
Leading knee-ankle	TCC	15.2 (5.3)	23.7 (12.9)	24.0 (5.5)	*p*_g_ = 0.04+	0.28
	Control	33.3 (15.9)	33.4 (14.9)	26.8 (10.8)	*p*_h_ = 0.62	0.04
Trailing hip-knee	TCC	4.2 (0.4)	3.4 (1.1)	4.4 (1.3)	*p*_g_ = 0.03+	0.21
	Control	4.6 (2.0)	4.7 (1.6)	5.5 (1.6)	*p*_h_ = 0.13	0.09
Trailing knee-ankle	TCC	9.5 (1.1)	11.5 (4.3)	15.7 (4.8)	*p*_g_ = 0.01+	0.61
	Control	15.6 (7.1)	20.0 (5.0)	17.2 (6.3)	*p*_h_ = 0.21	0.13
Leading limb SLS					
Leading hip-knee	TCC	6.7 (3.3)	5.8 (1.8)	4.9 (1.1)	*p*_g_ = 0.84	0.01
	Control	6.1 (1.8)	5.8 (4.0)	5.6 (3.0)	*p*_h_ = 0.72	0.03
Leading knee-ankle	TCC	13.3 (5.8)	17.5 (7.7)	15.8 (8.6)	*p*_g_ = 0.02+	0.37
	Control	21.0 (10.5)	26.2 (11.1)	27.5 (5.4)	*p*_h_ = 0.15	0.15
Trailing hip-knee	TCC	5.7 (1.2)	5.3 (1.6)	5.4 (2.1)	*p*_g_ = 0.01+	0.29
	Control	8.0 (1.5)	7.3 (2.6)	6.5 (1.8)	*p*_h_ = 0.10	0.10
Trailing knee-ankle	TCC	15.2 (6.0)	17.0 (7.0)	16.8 (6.2)	*p*_g_ = 0.02+	0.31
	Control	20.2 (4.6)	25.8 (11.0)	22.8 (9.9)	*p*_h_ = 0.34	0.07

LL, leg length; *ph*, *p*-value for obstacle height effect; *pg*, *p*-value for group effect; +, significant difference between groups; ↑, linearly increasing trend; ↓, linearly decreasing trend.

## Discussion

Ageing is often accompanied with impaired proprioception, deteriorated afferent and efferent systems, degraded central processing and a changed musculoskeletal system (Bougie and Morgenthal, 2001), affecting the performance of daily activities including obstacle-crossing (Chen et al., 1991; Hahn and Chou, 2004; Schillings et al., 2005; Lu et al., 2006). However, normal ageing did not seem to affect the lower limb inter-joint coordination patterns during obstacle-crossing as healthy older people were found to have inter-joint coordination patterns similar to those of healthy young controls (Yen et al., 2009). In the current study, the patterns of the joint phase plots indicate that the inter-segment coordination for each limb joint was largely independent of obstacle height during obstacle crossing for both groups. For the leading limb, TCC training altered the patterns and magnitudes of the hip-knee and knee-ankle coordination primarily over DLS, as indicated by the relatively low CMC values and high RMSD values (Figure 3; Table 3). The CRP curves of the knee-ankle coordination in the TCC group appeared to be closer to a more in-phase coordination pattern than for Control (Figure 3). For the trailing limb, TCC training mainly altered the knee-ankle coordination patterns and magnitudes throughout the crossing cycle, being closer to more in-phase coordination during SLS and Swing, but more out-of-phase coordination during DLS (Figure 4). These observed patterns and magnitudes of the inter-joint coordination of the lower limbs during obstacle-crossing, especially during DLS, appeared to correspond to the TCC basic movements, which enforce integrated flexion and extension, and exaggerate joint range of motion with a gentle challenge to balance (Wang et al., 2010). When moving forward during DLS, the leading knee flexes and the leading ankle dorsiflexes while the trailing knee extends and the trailing ankle dorsiflexes (Wang et al., 2010). Another interesting finding is that both groups showed similar CRP values when the leading and trailing toes were above the obstacle (Table 2). This may suggest that the inter-joint coordination at the crossing instances was already optimized in people without TCC experience, as these instances are critical for successful crossing (Lu et al., 2006).

The human body can be described as a complex neuro-controller coupled with a non-linear dynamic system (Kelso et al., 1981). Therefore, the variability of the movement trajectories generated by this system is a product of input disturbances, neuromuscular control, biomechanical dynamics (Rosen, 1970), as well as constraints imposed by environmental, organismic and task-related variables (Newell and Corcos, 1993). Healthy older people were found to cross obstacles with increased foot-obstacle clearances compared to young subjects, presumably to reduce the risk of tripping because the greater the foot-obstacle clearance, the less likely the foot hitting the obstacle (Lu et al., 2006). However, they did so with increased variability of lower-limb inter-joint coordination (Yen et al., 2009). Increasing the foot-obstacle clearance would need to lift the swing limb higher, which may increase the muscular work at the joints of the stance limb and thus the mechanical energy expenditure required for maintaining balance. Without changing the inter-joint coordination patterns, these increased mechanical challenges may contribute to the increased variability of the inter-joint coordination observed in the current healthy older adults. On the other hand, the older TCC practitioners were found to cross obstacles with increased foot-obstacle clearances via a less variable lower-limb inter-joint coordination, especially knee-ankle coordination. Given the reported benefits of long-term TCC training on muscle strength, balance, inter-joint coordination and postural control (e.g., (Lan et al., 2000; Wang et al., 2010; Birimoglu Okuyan and Bilgili, 2017)), the current results suggest that the older long-term TCC practitioners had the necessary muscle strength and ability of precision limb position control for the observed changes in the foot-obstacle clearances and inter-joint coordination, which required even less mechanical energy expenditure (Kuo et al., 2021).

The time-varying relationship between the trailing limb and the obstacle increased the difficulty in the control of the inter-joint coordination of the trailing swing limb owing to the lack of continuous visual feedback regarding the position of the obstacle. The variabilities of the inter-joint coordination of the trailing swing-limb were generally greater than those of the leading swing-limb, without significant differences between the young and old (Wang et al., 2009). The reduced variability of the knee-ankle CRP curves in both limbs might also result from better stability of the inter-joint coordination of the stance-limb (Lu et al., 2006). The TCC subjects also showed reduced variability of the swing hip-knee coordination during trailing-crossing, a major difference from the healthy controls (Lu et al., 2006). The results of the current study indicate that TCC training helped improve the repeatability of the movement control of the lower extremities, not only for the leading, but also for the trailing swing-limb during obstacle-crossing.

Previous studies showed that long-term practice of TCC helped increase the muscle strength in the lower extremities (Jacobson et al., 1997; Lan et al., 2000; Wu et al., 2002b) and that it can attenuate the general age decline in physical function (Tse and Bailey, 1992; Wolfson et al., 1996; Wolf et al., 1997; Hass et al., 2004). The observed inter-joint coordination strategies of obstacle-crossing in the TCC group, i.e., different patterns and magnitudes of inter-joint coordination during DLS, especially knee-ankle coordination, may be explained by the slow movement patterns emphasizing between-limb transfer of body weight in the TCC training. The results of the current study suggest that TCC practice was helpful for increasing the foot-obstacle clearances for both the leading and trailing limbs, with reduced risk of tripping, while with significantly reduced variability of the way the motions of the lower limb joints are coordinated during obstacle-crossing.

The current study was the first to compare the inter-joint coordination patterns and variability during obstacle-crossing between healthy older adults with long-term TCC experience and those without any TCC experience, identifying the effects of long-term TCC training. A cross-sectional study design was necessary for the current study involving older people who had practised TCC for more than 13 years. Such study design may involve uncertainties related to possible between-group differences at baseline and lack of data pre and post TCC training, preventing us from attributing all the observed between-group differences purely to long-term TCC practice. However, the confounding effects of these uncertainties on the outcome measures after 13 years may be much smaller than those for a relatively short TCC training period. Careful selection of the control group to match with the TCC group for sex, age, and BMI was also helpful for reducing the confounding effects. Moreover, the most plausible interpretations of the current findings were provided through discussions and comparisons with previous studies. Nonetheless, further longitudinal studies will be needed to overcome the limitations of a cross-sectional study and determine the effects of the TCC training period on the patterns and variability of the inter-joint coordination during obstacle-crossing in the elderly. On the other hand, the current study was limited to lower limb inter-joint coordination in the sagittal plane. Further investigation may include coordination between the trunk and the lower limb joints in all three planes, especially for pathological conditions that involve greater motions out of the sagittal plane. Further study may be needed to test whether TCC training would be helpful for older people with a higher risk of falling, such as those with underlying diseases that may compromise their balance control during gait.

## Conclusion

Older people with long-term TCC experience were found to exhibit specific inter-joint coordination patterns with increased foot-obstacle clearances for safer and more stable obstacle-crossing. For the leading limb, TCC training altered the patterns and magnitudes of the hip-knee and knee-ankle coordination primarily over DLS and significantly reduced the variabilities of knee-ankle coordination over the crossing cycle, regardless of obstacle height. For the trailing limb, TCC training altered mainly the patterns and magnitudes of the knee-ankle coordination with reduced variabilities. The patterns of the trailing hip-knee coordination over DLS were also changed, with reduced variabilities throughout the crossing cycle. The current results suggest that long-term TCC practice was helpful for a crossing strategy with significantly increased foot-obstacle clearances and reduced variability of the way the motions of the lower limb joints are coordinated during obstacle-crossing. These benefits may be explained by the long-lasting and continuous practice of the slow movement patterns emphasizing between-limb transfer of body weight in TCC.

## Data Availability

The original contributions presented in the study are included in the article/Supplementary Material, further inquiries can be directed to the corresponding author.

## References

[B1] Bhala R. P. O’Donnell J. Thoppil E. (1982). Ptophobia: Phobic Fear of Falling and its Clinical Management. Phys. Ther. 62 (2), 187–190. 10.1093/ptj/62.2.187 6120526

[B2] Birimoglu Okuyan C. Bilgili N. (2017). Effect of Tai Chi Chuan on Fear of Falling Balance and Physical Self-Perception in Elderly: A Randomised Controlled Trial. Turk Geriatri Dergisi 20, 232–241. https://reurl.cc/OkZx9A.

[B3] Birimoglu Okuyan C. Deveci E. (2021). The Effectiveness of Tai Chi Chuan on Fear of Movement, Prevention of Falls, Physical Activity, and Cognitive Status in Older Adults with Mild Cognitive Impairment: A Randomized Controlled Trial. Perspect. Psychiatr. Care 57 (3), 1273–1281. 10.1111/ppc.12684 33184928

[B4] Blake A. J. Morgan K. Bendall M. J. Dallosso H. Ebrahim S. B. J. Arie T. H. D. (1988). Falls by Elderly People at home: Prevalence and Associated Factors. Age Ageing 17 (6), 365–372. 10.1093/ageing/17.6.365 3266440

[B5] Bougie J. D. Morgenthal A. P. (2001). The Aging Body: Conservative Management of Common Neuromusculoskeletal Conditions. New York, NY: McGraw-Hill, Medical Pub Division.

[B6] Burgess-Limerick R. Abernethy B. Neal R. J. (1993). Relative Phase Quantifies Interjoint Coordination. J. Biomech. 26 (1), 91–94. 10.1016/0021-9290(93)90617-n 8423174

[B7] Burschka J. M. Keune P. M. Oy U. H. Oschmann P. Kuhn P. (2014). Mindfulness-Based Interventions in Multiple Sclerosis: Beneficial Effects of Tai Chi on Balance, Coordination, Fatigue and Depression. BMC Neurol. 14 (1), 165–169. 10.1186/s12883-014-0165-4 25145392 PMC4236646

[B8] Campbell A. J. Borrie M. J. Spears G. F. Jackson S. L. Brown J. S. Fitzgerald J. L. (1990). Circumstances and Consequences of Falls Experienced by a Community Population 70 Years and over during a Prospective Study. Age Ageing 19 (2), 136–141. 10.1093/ageing/19.2.136 2337010

[B9] Chang Y.-T. Huang C.-F. Chang J.-H. (2015). The Effect of Tai Chi Chuan on Obstacle Crossing Strategy in Older Adults. Res. Sports Med. 23 (3), 315–329. 10.1080/15438627.2015.1040920 26114218

[B10] Chen H.-c. Ashton-Miller J. A. Alexander N. B. Schultz A. B. (1994). Effects of Age and Available Response Time on Ability to Step over an Obstacle. J. Gerontol. 49 (5), M227–M233. 10.1093/geronj/49.5.m227 8056942

[B11] Chen H.-C. Ashton-Miller J. A. Alexander N. B. Schultz A. B. (1991). Stepping over Obstacles: Gait Patterns of Healthy Young and Old Adults. J. Gerontol. 46 (6), M196–M203. 10.1093/geronj/46.6.m196 1940078

[B12] Chen H.-L. Lu T.-W. Chou L.-S. (2015). Effect of Concussion on Inter-Joint Coordination During Divided-Attention Gait. J. Med. Biol. Eng. 35 (1), 28–33. 10.1007/s40846-015-0002-2

[B13] Chen H.-L. Lu T.-W. (2006). Comparisons of the Joint Moments Between Leading and Trailing Limb in Young Adults when Stepping over Obstacles. Gait Posture 23 (1), 69–77. 10.1016/j.gaitpost.2004.12.001 16311197

[B14] Chen H.-L. Lu T.-W. Lin H. C. (2004). Three-Dimensional Kinematic Analysis of Stepping Over Obstacles in Young Subjects. Biomed. Eng. Appl. Basis Commun. 16 (03), 157–164. 10.4015/s1016237204000219

[B15] Chen H.-L. Lu T.-W. Wang T.-M. Huang S.-C. (2008). Biomechanical Strategies for Successful Obstacle Crossing with the Trailing Limb in Older Adults with Medial Compartment Knee Osteoarthritis. J. Biomech. 41 (4), 753–761. 10.1016/j.jbiomech.2007.11.017 18177877

[B16] Chien H.-L. Lu T.-W. (2017). Effects of Shoe Heel Height on the End-Point and Joint Kinematics of the Locomotor System When Crossing Obstacles of Different Heights. Ergonomics 60 (3), 410–420. 10.1080/00140139.2016.1175672 27153344

[B17] Chiu S.-L. Lu T.-W. Chou L.-S. (2010). Altered Inter-joint Coordination during Walking in Patients with Total Hip Arthroplasty. Gait Posture 32 (4), 656–660. 10.1016/j.gaitpost.2010.09.015 20947354

[B18] Choi J. H. Moon J.-S. Song R. (2005). Effects of Sun-Style Tai Chi Exercise on Physical Fitness and Fall Prevention in Fall-Prone Older Adults. J. Adv. Nurs. 51 (2), 150–157. 10.1111/j.1365-2648.2005.03480.x 15963186

[B19] Chou L.-S. Draganich L. F. (1998). Increasing Obstacle Height and Decreasing Toe-Obstacle Distance Affect the Joint Moments of the Stance Limb Differently when Stepping over an Obstacle. Gait Posture 8 (3), 186–204. 10.1016/s0966-6362(98)00034-4 10200408

[B20] Cirstea M. C. Mitnitski A. B. Feldman A. G. Levin M. F. (2003). Interjoint Coordination Dynamics During Reaching in Stroke. Exp. Brain Res. 151 (3), 289–300. 10.1007/s00221-003-1438-0 12819841

[B21] Cole G. K. Nigg B. M. Ronsky J. L. Yeadon M. R. (1993). Application of the Joint Coordinate System to Three-Dimensional Joint Attitude and Movement Representation: A Standardization Proposal. J. Biomech. Eng. 115 (4A), 344–349. 10.1115/1.2895496 8309227

[B22] Di Fabio R. P. Kurszewski W. M. Jorgenson E. E. Kunz R. C. (2004). Footlift Asymmetry During Obstacle Avoidance in High-Risk Elderly. J. Am. Geriatr. Soc. 52 (12), 2088–2093. 10.1111/j.1532-5415.2004.52569.x 15571548

[B23] Draganich L. F. Kuo C. E. (2004). The Effects of Walking Speed on Obstacle Crossing in Healthy Young and Healthy Older Adults. J. Biomech. 37 (6), 889–896. 10.1016/j.jbiomech.2003.11.002 15111076

[B24] Erdfelder E. Faul F. Buchner A. (1996). GPOWER: A General Power Analysis Program. Behav. Res. Methods Instr. Comput. 28 (1), 1–11. 10.3758/bf03203630

[B25] Ghoussayni S. Stevens C. Durham S. Ewins D. (2004). Assessment and Validation of a Simple Automated Method for the Detection of Gait Events and Intervals. Gait Posture 20 (3), 266–272. 10.1016/j.gaitpost.2003.10.001 15531173

[B26] Graafmans W. C. Ooms M. E. Hofstee H. M. A. Bezemer P. D. Bouter L. M. Lips P. (1996). Falls in the Elderly: A Prospective Study of Risk Factors and Risk Profiles. Am. J. Epidemiol. 143 (11), 1129–1136. 10.1093/oxfordjournals.aje.a008690 8633602

[B27] Hackney M. E. Earhart G. M. (2008). Tai Chi Improves Balance and Mobility in People with Parkinson Disease. Gait Posture 28 (3), 456–460. 10.1016/j.gaitpost.2008.02.005 18378456 PMC2552999

[B28] Haddad J. M. Emmerik R. E. A. V. Whittlesey S. N. Hamill J. (2006). Adaptations in Interlimb and Intralimb Coordination to Asymmetrical Loading in Human Walking. Gait Posture 23 (4), 429–434. 10.1016/j.gaitpost.2005.05.006 16099160

[B29] Hahn M. E. Chou L.-S. (2004). Age-Related Reduction in Sagittal Plane Center of Mass Motion during Obstacle Crossing. J. Biomech. 37 (6), 837–844. 10.1016/j.jbiomech.2003.11.010 15111071

[B30] Hamill J. van Emmerik R. E. A. Heiderscheit B. C. Li L. (1999). A Dynamical Systems Approach to Lower Extremity Running Injuries. Clin. Biomech. 14 (5), 297–308. 10.1016/s0268-0033(98)90092-4 10521606

[B31] Hass C. J. Gregor R. J. Waddell D. E. Oliver A. Smith D. W. Fleming R. P. (2004). The Influence of Tai Chi Training on the center of Pressure Trajectory during Gait Initiation in Older Adults. Arch. Phys. Med. Rehabil. 85 (10), 1593–1598. 10.1016/j.apmr.2004.01.020 15468016

[B32] Ho T. J. Chen S. C. Hong S. W. Lu T. W. Lin J. G. (2012). Influence of Long-Term Tai-Chi Chuan Training on Standing Balance in the Elderly. Biomed. Eng. Appl. Basis Commun. 24 (1), 17–25. 10.4015/s1016237212002913

[B33] Hong S.-W. Leu T.-H. Wang T.-M. Li J.-D. Ho W.-P. Lu T.-W. (2015). Control of Body’s Center of Mass Motion Relative to Center of Pressure During Uphill Walking in the Elderly. Gait & Posture 42 (4), 523–528. 10.1016/j.gaitpost.2015.08.007 26386677

[B34] Hsu W.-C. Liu M.-W. Lu T.-W. (2016). Biomechanical Risk Factors for Tripping during Obstacle-Crossing with the Trailing Limb in Patients with Type II Diabetes Mellitus. Gait & Posture 45, 103–109. 10.1016/j.gaitpost.2016.01.010 26979890

[B35] Jacobson B. H. Ho-Cheng C. Cashel C. Guerrero L. (1997). The Effect of T'ai Chi Chuan Training on Balance, Kinesthetic Sense, and Strength. Percept Mot. Skills 84 (1), 27–33. 10.2466/pms.1997.84.1.27 9132718

[B36] Kadaba M. P. Ramakrishnan H. K. Wootten M. E. Gainey J. Gorton G. Cochran G. V. B. (1989). Repeatability of Kinematic, Kinetic, and Electromyographic Data in Normal Adult Gait. J. Orthop. Res. 7 (6), 849–860. 10.1002/jor.1100070611 2795325

[B37] Kim H.-D. (2009). Effects of Tai Chi Exercise on the Center of Pressure Trace During Obstacle Crossing in Older Adults who are at a Risk of Falling. J. Phys. Ther. Sci. 21 (1), 49–54. 10.1589/jpts.21.49

[B38] Kim H.-D. Je H. D. Jeong J. H. Ma S.-Y. (2013). Effects of Tai Chi Training on Obstacle Avoidance and Gait Initiation by Older People. J. Phys. Ther. Sci. 25 (2), 193–198. 10.1589/jpts.25.193

[B39] Kong Z. Sze T.-M. Yu J. Loprinzi P. Xiao T. Yeung A. (2019). Tai Chi as an Alternative Exercise to Improve Physical Fitness for Children and Adolescents with Intellectual Disability. Ijerph 16 (7), 1152. 10.3390/ijerph16071152 PMC6479776 30935071

[B40] Kuo C.-C. Chen S.-C. Wang J.-Y. Ho T.-J. Lu T.-W. (2021). Best-Compromise Control Strategy between Mechanical Energy Expenditure and Foot Clearance for Obstacle-Crossing in Older Adults: Effects of Tai-Chi Chuan Practice. Front. Bioeng. Biotechnol. 9, 774771. 10.3389/fbioe.2021.774771 34926422 PMC8675231

[B41] Lai J.-S. Lan C. Wong M.-K. Teng S.-H. (1995). Two-Year Trends in Cardiorespiratory Function Among Older Tai Chi Chuan Practitioners and Sedentary Subjects. J. Am. Geriatr. Soc. 43 (11), 1222–1227. 10.1111/j.1532-5415.1995.tb07397.x 7594155

[B42] Lan C. Lai J.-S. Chen S.-Y. Wong M.-K. (2000). Tai Chi Chuan to Improve Muscular Strength and Endurance in Elderly Individuals: a Pilot Study. Arch. Phys. Med. Rehabil. 81 (5), 604–607. 10.1016/s0003-9993(00)90042-x 10807099

[B43] Li F. Harmer P. Fitzgerald K. Eckstrom E. Stock R. Galver J. (2012). Tai Chi and Postural Stability in Patients with Parkinson’s Disease. N. Engl. J. Med. 366 (6), 511–519. 10.1056/nejmoa1107911 22316445 PMC3285459

[B44] Li F. Harmer P. Liu Y. Eckstrom E. Fitzgerald K. Stock R. (2014). A Randomized Controlled Trial of Patient‐Reported Outcomes with Tai Chi Exercise in Parkinson’s Disease. Mov Disord. 29 (4), 539–545. 10.1002/mds.25787 24375468 PMC3976742

[B45] Li L. van den Bogert E. C. H. Caldwell G. E. van Emmerik R. E. A. Hamill J. (1999). Coordination Patterns of Walking and Running at Similar Speed and Stride Frequency. Hum. Move. Sci. 18 (1), 67–85. 10.1016/s0167-9457(98)00034-7

[B46] Lin M.-R. Hwang H.-F. Wang Y.-W. Chang S.-H. Wolf S. L. (2006). Community-Based Tai Chi and its Effect on Injurious Falls, Balance, Gait, and Fear of Falling in Older People. Phys. Ther. 86 (9), 1189–1201. 10.2522/ptj.20040408 16959668

[B47] Liu H.-H. Yeh N.-C. Wu Y.-F. Yang Y.-R. Wang R.-Y. Cheng F.-Y. (2019). Effects of Tai Chi Exercise on Reducing Falls and Improving Balance Performance in Parkinson’s Disease: A Meta-Analysis. Parkinson’s Dis. 2019, 1–18. 10.1155/2019/9626934 PMC6409066 30918623

[B48] Liu M.-W. Hsu W.-C. Lu T.-W. Chen H.-L. Liu H.-C. (2010). Patients with Type II Diabetes Mellitus Display Reduced Toe-Obstacle Clearance with Altered Gait Patterns during Obstacle-Crossing. Gait Posture 31 (1), 93–99. 10.1016/j.gaitpost.2009.09.005 19875290

[B49] Lu T.-W. Chen H.-L. Chen S.-C. (2006). Comparisons of the Lower Limb Kinematics between Young and Older Adults when Crossing Obstacles of Different Heights. Gait Posture 23 (4), 471–479. 10.1016/j.gaitpost.2005.06.005 16023346

[B50] Lu T.-W. Chen H.-L. Wang T.-M. (2007). Obstacle Crossing in Older Adults with Medial Compartment Knee Osteoarthritis. Gait Posture 26 (4), 553–559. 10.1016/j.gaitpost.2006.12.002 17240144

[B51] Lu T.-W. O’Connor J. J. (1999). Bone Position Estimation from Skin Marker Co-ordinates Using Global Optimisation with Joint Constraints. J. Biomech. 32 (2), 129–134. 10.1016/s0021-9290(98)00158-4 10052917

[B52] Lu T.-W. Yen H.-C. Chen H.-L. (2008). Comparisons of the Inter-Joint Coordination Between Leading and Trailing Limbs when Crossing Obstacles of Different Heights. Gait Posture 27 (2), 309–315. 10.1016/j.gaitpost.2007.04.007 17499992

[B53] Lu T.-W. Yen H.-C. Chen H.-L. Hsu W.-C. Chen S.-C. Hong S.-W. (2010). Symmetrical Kinematic Changes in Highly Functioning Older Patients Post-Stroke During Obstacle-Crossing. Gait Posture 31 (4), 511–516. 10.1016/j.gaitpost.2010.02.012 20299223

[B54] Mak M. K. Ng P. L. (2003). Mediolateral Sway in Single-Leg Stance is the Best Discriminator of Balance Performance for Tai-Chi Practitioners. Arch. Phys. Med. Rehabil. 84 (5), 683–686. 10.1016/s0003-9993(03)04810-4 12736881

[B55] Mak M. K. Wong-Yu I. S. Shen X. Chung C. L. (2017). Long-Term Effects of Exercise and Physical Therapy in People with Parkinson Disease. Nat. Rev. Neurol. 13 (11), 689–703. 10.1038/nrneurol.2017.128 29027544

[B56] McFadyen B. J. Prince F. (2002). Avoidance and Accommodation of Surface Height Changes by Healthy, Community-Dwelling, Young, and Elderly Men. J. Gerontol. Ser. A: Biol. Sci. Med. Sci. 57 (4), B166–B174. 10.1093/gerona/57.4.b166 11909882

[B57] McKenzie N. C. Brown L. A. (2004). Obstacle Negotiation Kinematics: Age-dependent Effects of Postural Threat. Gait Posture 19 (3), 226–234. 10.1016/S0966-6362(03)00060-2 15125911

[B58] Newell K. Corcos D. (1993). Variability and Motor Control. Champaign: Human Kinetics.

[B59] Overstall P. W. Exton-Smith A. N. Imms F. J. Johnson A. L. (1977). Falls in the Elderly Related to Postural Imbalance. Br. Med. J. 1 (6056), 261–264. 10.1136/bmj.1.6056.261 837061 PMC1604147

[B60] Peters B. T. Haddad J. M. Heiderscheit B. C. Van Emmerik R. E. A. Hamill J. (2003). Limitations in the Use and Interpretation of Continuous Relative Phase. J. Biomech. 36 (2), 271–274. 10.1016/s0021-9290(02)00341-x 12547366

[B61] Ramachandran A. K. Rosengren K. S. Yang Y. Hsiao-Wecksler E. T. (2007). Effect of Tai Chi on Gait and Obstacle Crossing Behaviors in Middle-Aged Adults. Gait & Posture 26 (2), 248–255. 10.1016/j.gaitpost.2006.09.005 17035021

[B62] Rosen R. (1970). Dynamical System Theory in Biology. New York: Wiley-Interscience.

[B63] Schillings A. M. Mulder T. Duysens J. (2005). Stumbling Over Obstacles in Older Adults Compared to Young Adults. J. Neurophysiol. 94 (2), 1158–1168. 10.1152/jn.00396.2004 15615837

[B64] Scott Kelso J. A. Holt K. G. Rubin P. Kugler P. N. (1981). Patterns of Human Interlimb Coordination Emerge from the Properties of Non-Linear, Limit Cycle Oscillatory Processes. J. Mot. Behav. 13 (4), 226–261. 10.1080/00222895.1981.10735251 23962314

[B65] Silfies S. P. Bhattacharya A. Biely S. Smith S. S. Giszter S. (2009). Trunk Control during Standing Reach: A Dynamical System Analysis of Movement Strategies in Patients with Mechanical Low Back Pain. Gait Posture 29 (3), 370–376. 10.1016/j.gaitpost.2008.10.053 19046882 PMC2671394

[B66] Stergiou N. Jensen J. L. Bates B. T. Scholten S. D. Tzetzis G. (2001a). A Dynamical Systems Investigation of Lower Extremity Coordination During Running Over Obstacles. Clin. Biomech. 16 (3), 213–221. 10.1016/s0268-0033(00)00090-5 11240056

[B67] Stergiou N. Scholten S. D. Jensen J. L. Blanke D. (2001b). Intralimb Coordination Following Obstacle Clearance During Running: the Effect of Obstacle Height. Gait Posture 13 (3), 210–220. 10.1016/s0966-6362(00)00101-6 11323227

[B68] Tinetti M. E. Speechley M. (1989). Prevention of Falls Among the Elderly. N. Engl. J. Med. 320 (16), 1055–1059. 10.1056/NEJM198904203201606 2648154

[B69] Tinetti M. E. De Leon C. F. M. Doucette J. T. Baker D. I. (1994). Fear of Falling and Fall-Related Efficacy in Relationship to Functioning Among Community-Living Elders. J. Gerontol. 49 (3), M140–M147. 10.1093/geronj/49.3.m140 8169336

[B70] Tinetti M. E. Speechley M. Ginter S. F. (1988). Risk Factors for Falls Among Elderly Persons Living in the Community. N. Engl. J. Med. 319 (26), 1701–1707. 10.1056/nejm198812293192604 3205267

[B71] Tsang W. W. N. Hui-Chan C. W. Y. (2004). Effect of 4- and 8-wk Intensive Tai Chi Training on Balance Control in the Elderly. Med. Sci. Sports Exerc. 36 (4), 648–657. 10.1249/01.mss.0000121941.57669.bf 15064593

[B72] Tsang W. W. Wong V. S. Fu S. N. Hui-Chan C. W. (2004). Tai Chi Improves Standing Balance Control Under Reduced or Conflicting Sensory Conditions. Arch. Phys. Med. Rehabil. 85 (1), 129–137. 10.1016/j.apmr.2003.02.002 14970980

[B73] Tse S.-K. Bailey D. M. (1992). T'ai Chi and Postural Control in the Well Elderly. Am. J. Occup. Ther. 46 (4), 295–300. 10.5014/ajot.46.4.295 1566796

[B74] van Uden C. J. T. Bloo J. K. C. Kooloos J. G. M. van Kampen A. de Witte J. Wagenaar R. C. (2003). Coordination and Stability of One-Legged Hopping Patterns in Patients with Anterior Cruciate Ligament Reconstruction: Preliminary Results. Clin. Biomech. 18 (1), 84–87. 10.1016/s0268-0033(02)00170-5 12527252

[B75] Vellas B. J. Wayne S. J. Romero L. J. Baumgartner R. N. Garry P. J. (1997). Fear of Falling and Restriction of Mobility in Elderly Fallers. Age Ageing 26 (3), 189–193. 10.1093/ageing/26.3.189 9223714

[B76] Wang L.-H. Lo K.-C. Lin C.-J. Su F.-C. (2010). Multijoint Coordination of Lower Extremity in Tai Chi Exercise. J. Mech. Med. Biol. 10 (03), 479–493. 10.1142/s0219519410003526

[B77] Wang T.-M. Yen H.-C. Lu T.-W. Chen H.-L. Chang C.-F. Liu Y.-H. (2009). Bilateral Knee Osteoarthritis Does Not Affect Inter-joint Coordination in Older Adults With Gait Deviations During Obstacle-Crossing. J. Biomech. 42 (14), 2349–2356. 10.1016/j.jbiomech.2009.06.029 19679309

[B78] Weerdesteyn V. Rijken H. Geurts A. C. H. Smits-Engelsman B. C. M. Mulder T. Duysens J. (2006). A Five-Week Exercise Program can Reduce Falls and Improve Obstacle Avoidance in the Elderly. Gerontology 52 (3), 131–141. 10.1159/000091822 16645293

[B79] Winter D. A. (1992). Foot Trajectory in Human Gait: A Precise and Multifactorial Motor Control Task. Phys. Ther. 72 (1), 45–53. 10.1093/ptj/72.1.45 1728048

[B80] Wolf S. L. Barnhart H. X. Ellison G. L. Coogler C. E. (1997). The Effect of Tai Chi Quan and Computerized Balance Training on Postural Stability in Older Subjects. Phys. Ther. 77 (4), 371–381. 10.1093/ptj/77.4.371 9105340

[B81] Wolfson L. Whipple R. Derby C. Judge J. King M. Amerman P. (1996). Balance and Strength Training in Older Adults: Intervention Gains and Tai Chi Maintenance. J. Am. Geriatr. Soc. 44 (5), 498–506. 10.1111/j.1532-5415.1996.tb01433.x 8617896

[B82] Woltring H. J. (19861978). A Fortran Package for Generalized, Cross-Validatory Spline Smoothing and Differentiation. Adv. Eng. Softw. 8 (2), 104–113. 10.1016/0141-1195(86)90098-7

[B83] Wong A. M. K. Pei Y.-C. Lan C. Huang S.-C. Lin Y.-C. Chou S.-W. (2009). Is Tai Chi Chuan Effective in Improving Lower Limb Response Time to Prevent Backward Falls in the Elderly? Age 31 (2), 163–170. 10.1007/s11357-009-9094-3 19415525 PMC2693735

[B84] Wong A. M. Lin Y.-C. Chou S.-W. Tang F.-T. Wong P.-Y. (2001). Coordination Exercise and Postural Stability in Elderly People: Effect of Tai Chi Chuan. Arch. Phys. Med. Rehabil. 82 (5), 608–612. 10.1053/apmr.2001.22615 11346836

[B85] Wu G. (2002). Evaluation of the Effectiveness of Tai Chi for Improving Balance and Preventing Falls in the Older Populationâ€"A Review. J. Am. Geriatr. Soc. 50 (4), 746–754. 10.1046/j.1532-5415.2002.50173.x 11982679

[B86] Wu G. Siegler S. Allard P. Kirtley C. Leardini A. Rosenbaum D. (2002a). ISB Recommendation on Definitions of Joint Coordinate System of Various Joints for the Reporting of Human Joint Motion-Part I: Ankle, Hip, and Spine. J. Biomech. 35 (4), 543–548. 10.1016/s0021-9290(01)00222-6 11934426

[B87] Wu G. Zhao F. Zhou X. Wei L. (2002b). Improvement of Isokinetic Knee Extensor Strength and Reduction of Postural Sway in the Elderly from Long-Term Tai Chi Exercise. Arch. Phys. Med. Rehabil. 83 (10), 1364–1369. 10.1053/apmr.2002.34596 12370869

[B88] Wu K.-W. Li J.-D. Huang H.-P. Liu Y.-H. Wang T.-M. Ho Y.-T. (2019). Bilateral Asymmetry in Kinematic Strategies for Obstacle-Crossing in Adolescents with Severe Idiopathic Thoracic Scoliosis. Gait Posture 71, 211–218. 10.1016/j.gaitpost.2019.05.007 31078825

[B89] Yen H.-C. Chen H.-L. Liu M.-W. Liu H.-C. Lu T.-W. (2009). Age Effects on the Inter-joint Coordination during Obstacle-Crossing. J. Biomech. 42 (15), 2501–2506. 10.1016/j.jbiomech.2009.07.015 19665128

